# Using *c-kit* to genetically target cerebellar molecular layer interneurons in adult mice

**DOI:** 10.1371/journal.pone.0179347

**Published:** 2017-06-28

**Authors:** Samantha B. Amat, Matthew J. M. Rowan, Michael A. Gaffield, Audrey Bonnan, Chikako Kikuchi, Hiroki Taniguchi, Jason M. Christie

**Affiliations:** Max Planck Florida Institute for Neuroscience, Jupiter, FL, United States of America; Institut de la vision, FRANCE

## Abstract

The cerebellar system helps modulate and fine-tune motor action. Purkinje cells (PCs) provide the sole output of the cerebellar cortex, therefore, any cerebellar involvement in motor activity must be driven by changes in PC firing rates. Several different cell types influence PC activity including excitatory input from parallel fibers and inhibition from molecular layer interneurons (MLIs). Similar to PCs, MLI activity is driven by parallel fibers, therefore, MLIs provide feed-forward inhibition onto PCs. To aid in the experimental assessment of how molecular layer inhibition contributes to cerebellar function and motor behavior, we characterized a new knock-in mouse line with Cre recombinase expression under control of endogenous *c-kit* transcriptional machinery. Using these engineered c-Kit mice, we were able to obtain high levels of conditional MLI transduction in adult mice using Cre-dependent viral vectors without any PC or granule cell labeling. We then used the mouse line to target MLIs for activity perturbation *in vitro* using opto- and chemogenetics.

## Introduction

Neural activity in the cerebellum contributes to the coordination of motor tasks and is required for adaptation of many learned movements [1–4]. A single neuron type, the Purkinje cell (PC), provides the sole output from the cerebellar cortex. Therefore, elucidation of cerebellar function during movement requires an understanding of how the various inputs onto PCs affect their excitability and spike output. Most prominently, these include both excitatory climbing fiber projections from the inferior olive and parallel fiber axons of granule cells (GrCs) as well as two types of inhibitory molecular layer interneurons: stellate cells and basket cells (collectively referred to as MLIs). Experimental approaches to reveal the role of cell types in neural circuit function are greatly aided by targeting strategies often involving conditional mutagenesis using the Cre-loxP recombination system under cell-type control of a genetic marker [5]. For this reason, genetic targeting of MLIs is critical to advance circuit-level understanding of the cerebellum.

Genetic targeting of MLIs is particularly problematic, because PCs, like MLIs, are GABA-releasing and express many of the same genes commonly used to distinguish interneurons from projection neurons. For example, transcripts encoding for parvalbumin (PV) and Gad2 are present in both PCs and MLIs [6, 7]. An alternative MLI target, *Nos1*, encoding neuronal nitric oxide synthase (nNOS), avoids PCs but is found in GrCs, the source of the parallel fiber input to both PCs and MLIs [8–10]. Viral vectors, including recombinant adeno-associated virus (AAV), are often used to deliver transgenes in neural tissue and, when combined with Cre-activation in knock-in mice, offer excellent selectivity to drive gene expression in a cell-specific manner [11]. In addition, by capitalizing on the viral serotype tropism and the specificity of the transgene’s *cis*-regulatory elements (enhancers and promoters), viral platforms alone can achieve high heterogeneity with respect to cell type, including in the cerebellum [12–14]. However, virus-based strategies may add considerable experimental constraints if a specific viral serotype and promoter combination is required, for example, as when strong promotion is necessary for *in vivo* use of genetically-encoded reporter and effector proteins that facilitate measurement and manipulation of cell activity (e.g. calcium indicators).

Here we present data using a putative MLI target gene, *c-kit*, that encodes a receptor tyrosine kinase and proto-oncogene (c-Kit) expressed by MLIs but not mature PCs [15–18]. Instead, PCs express the endogenous ligand for c-Kit [15–17] making *c-kit* a good candidate gene to molecularly target MLIs. Indeed, mice carrying c-Kit-GFP reporter constructs show expression in MLIs [19, 20]. Using genetic engineering in mice, we developed a *c-kit*^*IRES-Cre*^ knock-in driver line allowing Cre-dependent transduction of MLIs with a variety of strong viral promoters in adult animals. Our anatomical and functional assessments showed no PC or GrC labeling and only minimal targeting of cells outside the molecular layer, as expected for the endogenous pattern of c-Kit expression in Golgi cells (GoCs) [15].

## Methods

### Mice

All animal procedures were approved by the Institute Animal Care and Use Committee at Max Planck Florida Institute for Neuroscience. Experiments were performed using either newborn (postnatal day [PND] 0) or mature (>PND 30) mice (25 total: 10 female, 15 male). The following mice line was from Jackson Labs: B6.Cg-*Gt(ROSA)26Sor*^*tm14(CAG-tdTomato)Hze*^*/*J (Ai14); stock #007914; [21]. Experiments were performed using heterozygous *c-kit*^*IRES-Cre*^ mice (PND 39–119). To examine tissue expression in the *c-kit*^*IRES-Cre*^ line, mice were bred to homozygous *loxP*-flanked tdTomato reporter (Ai14) mice.

For the *c-kit*^*IRES-Cre*^ line, a PCR-based cloning strategy was used to generate a knock-in vector. A BAC clone containing a *c-kit* genomic sequence (RP23-142L11) was used as a PCR template to amplify 5’ (~5 kb) and 3’ (~2kb) homology arm. The 5’ arm was cloned into PL450 containing an *IRES-Cre* cassette and an *frt-Neo-frt* cassette so that the stop codon of a *c-kit* gene is immediately followed by an *IRES-Cre* (PL450-5’ arm). The 3’ arm was cloned into PL253 including an *HSV-TK* gene, which is used for negative selection of ES cells (PL253-3’ arm). PL450-5’ arm and PL253-3’ arm vectors were cut with restriction enzymes (NotI/SacII) and fragments were ligated to generate a knock-in vector containing 5’ arm-(*IRES-Cre*)-(*frt-Neo-frt*)-3’ arm-(*HSV-TK*). Knock-in vectors were linearized by SwaI and transfected into 129SVj/B6 F1 hybrid ES cells (V6.5). Correctly targeted clones were selected by positive and negative selection with neomycin and gancyclovir, respectively. The selected clones were further screened by PCR using an internal primer and an external primer corresponding to a nucleotide sequence from a knock-in vector and a genomic region downstream to 3’ arm, respectively. Twelve out of 48 clones (25%) were positive in PCR screening. Five out of 12 positives were further tested by Southern blotting using probes against *Cre* and a genomic region downstream to 3’ arm. Genomic DNA was prepared, cut with HpaI, run on a gel, and transferred onto membranes. In 4 out of 5 clones, correctly targeted bands were detected with both probes. One of positive clones was used for tetraploid complementation to obtain male heterozygous mice following standard procedures. The resulting *c-kit*^*IRES-Cre*^ mice were bred into a Bl6/J background for ≥4 generations. Homozygous *c-kit*^*IRES-Cre*^ mice were healthy, breed well, and had no obvious phenotypes.

The presence of Cre in experimental *c-kit*^*IRES-Cre*^ mice was determined by PCR using the REDExtract-N-Amp Tissue Kit (Sigma, St. Louis, MO) and the following primers: Cre 1568 F (forward) 5’-CGG TCG ATG CAA CGA GTG ATG-3’ and Cre 1569 R (reverse) 5’- AGC CTG TTT TGC ACG TTC ACC-3’ (Eurofins Genomics, Louisville, KY).

### Surgical procedures

The following adeno-associated viruses (AAVs) were purchased from the University of Pennsylvania Vector Core (UPenn) or the University of North Carolina Vector Core (UNC): AAV1.CAG.Flex.GFP.WPRE.SV40 (UNC), AAV1.EF1α.DIO.eYFP.WPRE.hGH (UPenn), AAV1.hSyn.DIO.eGFP.WPRE.hGH (UPenn), AAV1.EF1α.DIO.hChR2(H134R).eYFP.WPRE.hGH (UPenn), AAV5.hSyn.DIO.hM4Di.mCherry.WPRE.hGH (UNC) and AAV8.EF1α.DIO.hM4Di.mCherry.WPRE.hGH (UNC). AAV1.CAG.Flex.ChR2.HA.2a.hM4Di was custom built and packaged by Vigene Biosciences (Rockville, MD). Undiluted viral solution was injected into mice from high titer stocks (≥ 10^12^ vg/ml) except for the bicistronic hM4Di construct injected in a 1:1 dilution with AAV1.CAG.Flex.GFP.WPRE.SV40 (≥ 10^11^ vg/ml) to visually identify transduced regions for electrophysiology.

For adult mice, viral injections were performed under isoflurane anesthesia (1.2–2% by volume in O_2_) using a stereotactic apparatus (David Kopf Instruments, Tujunga, CA). Depth of anesthesia was tested at regular intervals with a toe pinch. Mice were maintained at 37°C by a heating pad with a rectal thermometer providing biofeedback. Carprofen (5 mg/kg) and buprenorphine (1 mg/kg) were administered to reduce swelling and provide post-operative pain management. Once under anesthesia, the surgical area was cleared of fur with a depilatory cream, cleaned with a surgical scrub, and then a cocktail of lidocaine and bupivacaine was injected subcutaneously. A sagittal opening exposing the posterior part of the skull was made and a small hole was carefully drilled (or picked) over the cerebellar vermis (lobules IV-VI; from lambda: 0–0.6 mm right, 2–2.9 mm caudal, and 0.3–1.0 mm depth for histology) or hemisphere (Crus I/II; from lambda: 2.8–3.5 mm left or right, 1.8–2.4 mm caudal, and 0.35–1.4 mm depth for histology and electrophysiology). Stereotactic coordinates ranged in value in order to test the consistency of the expression and was similar in all regions injected. A beveled glass micropipette (30–40 μm diameter) was slowly inserted into the surface of the cerebellum. A picospritzer (Parker, Hollis, NH) was used to precisely control the rate and amount of injected material (23 psi, 10 ms pulses every 2.5 s). After injecting the full volume of virus (~0.25 μL/location), the glass micropipette was left in place for 10 minutes before withdrawal. Subsequently, the scalp was sutured and mice were monitored for any discomfort during surgical recovery until ambulatory.

For virus injections in newborns, pups were anesthetized by hypothermia. On the day of birth, pups were removed from the mother as an entire litter and held over a heating pad (~37°C). A single pup was placed in a latex sleeve to provide thermal protection to the skin and then immersed in an ice bath for 7 minutes to achieve a surgical plane of anesthesia that lasted the duration of the surgery (≤10 minutes). After induction of anesthesia, pups were moved to a stereotactic apparatus and a small opening was created in the skin caudal to vascular lambda using microscissors. A beveled glass micropipette was slowly inserted through the soft skull and 0.5 μL of virus was injected using a picospritzer. Three minutes after injection, the glass micropipette was slowly retracted. Mice were brought back to a physiological temperature on a heating pad and monitored for recovery. Once all pups were injected and recovered, they were returned to the mother as a group.

### Histology

One to four weeks after virus injection, mice were deeply anesthetized with a cocktail of ketamine (100mg/kg) and xylazine (10 mg/kg) and transcardially perfused with 0.1 M phosphate buffer (PB) followed by cold paraformaldehyde (4% in volume by PB). The cerebellum and brainstem were collected and post-fixed overnight at 4°C. Tissue was then washed with PB, embedded in 4% agar and sliced at 100 μm thick sections. Sagittal sections were collected in PB and mounted onto glass slides using anti-fade mounting media (Thermofisher Scientific, Waltham, MA). Care was taken to assay and compare reporter gene expression at similar locations relative to injection sites. Immunohistochemistry was used to label GoCs, oligodendrocytes or HA-tagged cells using previously characterized antibodies. For this procedure, cerebellar slices were first incubated in blocking solution (10% normal goat serum and 0.2% Titron X-100 in PB) for 1 hour at room temperature (23–25°C), followed by overnight incubation at 4°C in primary antibodies diluted in blocking solution. Primary antibodies included: rabbit anti-mGluR2/3 (1:1,000, #ab6438, Abcam, Cambridge, MA) for GoCs, mouse anti-Olig2 (1:300, #MABN50, Millipore, Billerica, MA) to label oligodendrocytes and rabbit anti-HA for the bicistronic ChR2-HM4Di virus (1:500, #ab9110, Abcam, Cambridge, MA). Sections were then rinsed three times in PB and incubated in secondary antibodies: either anti-rabbit AlexaFluor 633 for mGluR2/3 (1:1,000, #A-21070, Thermofisher Scientific), anti-mouse AlexaFluor 633 for Olig2 (1:300, #A-21136, Thermofisher Scientific) or anti-rabbit AlexaFluor 488 (1:500; #A-11034, Thermofisher Scientific) for the HA tag. Afterwards, slices were rinsed three times in PB and mounted onto glass slides using anti-fade mounting media.

Sections processed for PV staining were first treated in pepsin (5 mg/mL in 5 mM HCl) for 1 hour then washed three times in PB prior to incubation in blocking solution (10% normal goat serum and 1% Titron X-100 in PB). Subsequent steps were followed as described above using anti-PV (1:1,000, #ab11427, Abcam, Cambridge, MA) and anti-rabbit AlexaFluor 633 (1:1,000, #A-21136, Thermofisher Scientific) as the secondary antibody.

### Imaging

Fluorescence images were acquired with a Zeiss LSM 780 or a LSM 880 confocal scanning microscope with excitation from an argon (458, 488 and 514 nm) or HeNe laser (633 nm) using the following objectives: Plan Apochromat 20x (0.8 NA/air), Plan Apochromat 40x (1.3 NA/oil), C-Apochromat 40x (1.2 NA/water) or LCI Plan-Neofluar 63x (1.3 NA/multi-immersion). Using ZEN lite software (Zeiss), Z-stacks (typically ~7 μm total thickness when antibodies were used, ~30 μm thick otherwise) were transformed into single maximum intensity projection images and intensity levels were adjusted to display a dynamic range between the minimum and maximum pixel values in the image. Images were then cropped to illustrate a typical region using Photoshop (Adobe).

Intensity profiles were calculated using ImageJ software (NIH) from straight sections of cerebellar cortex (~300–400 μm) and averaging along the dimension parallel to the cortical layers. Cell body intensities were measured in ImageJ by first thresholding images to isolate cell bodies, then calculating the average pixel intensity for each ROI greater than a specific number of pixels (varied depending on the image resolution, typically ~5 μm^2^ of total area).

Cell density in *c-kit*^*IRES-Cre*^ mice was assessed in 10 sections from 4 mice (PND 54–141). The molecular layer and GrC layer were outlined and cells within each layer were identified by fluorescence (GFP/eYFP) and counted manually, then divided by the volume contained within the outlined stack. Quantification of cell numbers transduced in *c-kit*^*IRES-Cre*^ mice using immunolabeling was performed on fixed tissue from animals two weeks after virus injection. Only cells in the molecular layer were counted using a PV antibody. Cells were sorted into three groups: either double positive for GFP and PV, positive for GFP alone, or positive for PV alone. Data included 8 sections from 2 mice (PND 119). GoCs were identified using an mGluR2/3 antibody. Cells were sorted into two groups: double positive for both GFP and mGluR2/3 or mGluR2/3 positive only. Data included 9 sections from 7 mice (PND 39–63). All counting was performed manually.

### Electrophysiology

Acute parasagittal slices from cerebellar hemispheres were prepared from injected mice (>6 weeks of age; 2–4 weeks after viral injection) of either sex. Cerebella were removed from mice after decapitation under deep isoflurane anesthesia. Brain slices (200 μm) were sectioned using a vibroslicer in an ice-cold solution containing (in mM) 87 NaCl, 25 NaHO_3_, 2.5 KCl, 1.25 NaH_2_PO_4_, 2 MgCl_2_, 1 CaCl_2_, 10 glucose, and 7 sucrose. Slices were transferred to an incubation chamber containing (in mM) 128 NaCl, 26.2 NaHO_3_, 2.5 KCl, 1 NaH_2_PO_4_, 1.5 CaCl_2_, 1.5 MgCl_2_ and 11 glucose and maintained at 34°C for 40 min and then at room temperature (23–25°C) until use. Slices were placed in a submersion chamber and continuously superfused with the same solution at 32°C. All solutions were oxygenated with carbogen gas (95% O_2_, 5% CO_2_) to equilibrium. Where noted, GABA_A_, NMDA, and AMPA receptors were blocked with (in μM) 100 picrotoxin, 10 *r*-CPP, and 10 NBQX (Tocris, Bristol, UK), respectively. Cells were targeted for patching using gradient-contrast infrared video microscopy. For whole-cell recording, pipettes (4–6 MΩ) were filled with either (in mM) 124 potassium gluconate, 2 KCl, 9 HEPES, 4 MgCl_2_, 4 NaATP, 3 L-Ascorbic Acid and 0.5 NaGTP for ChR2 photostimulation experiments or 120 CH_3_SO_3_H, 10 HEPES, 7 KCl, 4 Na_2_ATP, 0.5 Na_2_GTP, 2 MgCl_2_, 3 L-Ascorbic Acid for chemogenetic experiments. CNO (Tocris) was dissolved in DMSO to 50 mM, then diluted in the bath solution to the final working concentration.

A Multiclamp 700B amplifier (Molecular Devices, Sunnyvale, CA) was used for electrophysiological recording. Analog signals were low-pass filtered at 2–10 kHz and digitized at 20–50 kHz (Digidata 1440A, Molecular Devices) using pClamp software (Molecular Devices). Cells were always patched in an area with dense expression as determined by visual inspection using epi-fluorescence imaging (eYFP, mCherry or GFP for optogenetic and chemogenetic approaches, respectively). Notably, our recordings were not biased in that we did not specifically target individual fluorescent cells (i.e., cells were randomly selected within the field-of-view). Cell types were identified using gradient contrast imaging based on their size and position in the cerebellar cortex, with the exception of GoCs. For GoC recordings, Alexa 594 (60 μM) was included in the patch pipette to confirm their identity based on their unique morphology by imaging with two-photon microscopy during an experiment. GoCs were also distinguished based on their capacitance (~30 pF; [22]) with respect to GrCs (~5 pF; [23]) found in the same layer.

For optogenetic stimulation, brief light pulses (470 nm) were directed through a 60x (1.0 NA) immersion objective from an LED source. In current clamp experiments, continuous bias current was injected to hold cells at ~ -70 mV, except in a subset of PC recordings where a suprathreshold bias current was applied such that cells fired in a sustained manner. For direct electrical stimulation of MLIs, a second electrode containing ACSF was placed in the molecular layer. Brief electrical stimuli (200 μs pulses; 0.2 Hz) elicited IPSCs in PCs with bath perfusion of *r*-CPP and NBQX (picrotoxin omitted) to block parallel fiber-mediated excitatory transmission. In a similar manner, we used electrical stimulation (200 μs pulses; 100 Hz) from an electrode placed in the GrC layer to elicit GABA release from putative GoCs. This allowed us to evaluate for the effect of inhibition on GrC firing induced by direct current injection. For these experiments, excitatory transmission was blocked by NBQX (10 μM); electrical stimulation occurred over the duration of GrC depolarization (200 ms) in interleaved control trials which lacked extracellular stimulation. For each condition in our electrophysiological experiments, we typically averaged responses over 40 trials.

### Analysis

Axograph X, Graphpad Prism (Graphpad Software) and Excel (Microsoft) were used for analysis with values in text and figures reported as mean (± SEM) unless noted otherwise. Statistical differences were deemed significant with α values of p < 0.05. Paired t-tests were used for matched parametric datasets. Normality was determined using D’Agostino & Pearson omnibus or Shapiro-Wilk tests. Plots were created in Graphpad Prism and figures generated in Adobe Illustrator.

## Results

### MLI targeting using *c-kit*^*IRES-Cre*^ mice

**Fig 1:**
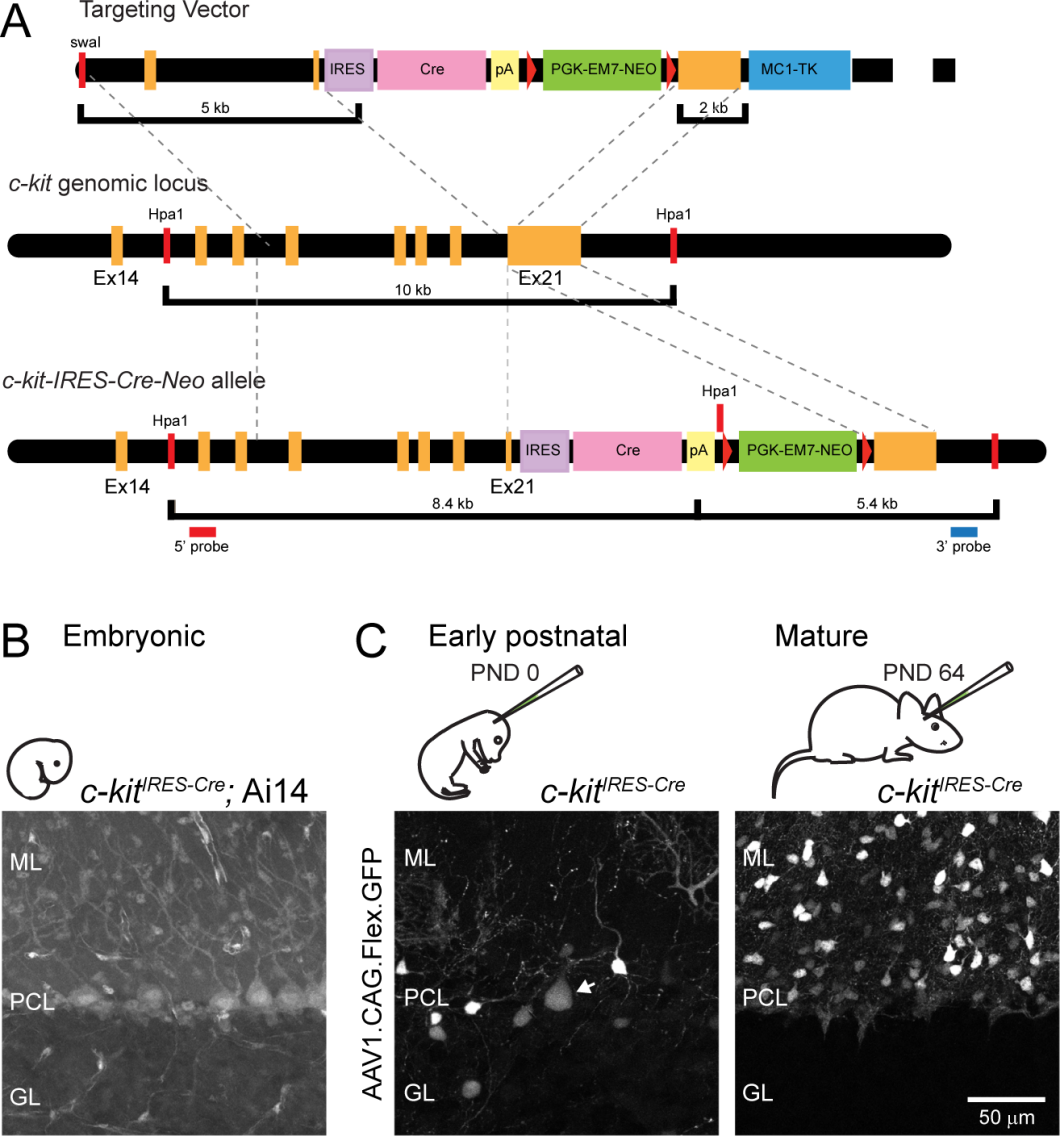
*c-kit*^*IRES-Cre*^ mice allow for MLI targeting in mature mice. **(A)** Schematic of *c-kit*^*IRES-Cre*^ mouse generation. ML, molecular layer; PCL, Purkinje cell layer; GL, granule cell layer. **(B)** Reporter protein expression in a *c-kit*^*IRES-Cre*^:Ai14 (*lox*-P flanked, Rosa26-tdTomato) mouse perfused at PND 38. This cross will report the entire history of Cre recombinase activity including during embryogenesis. **(C)** The cerebella from *c-kit*^*IRES-Cre*^ mice injected with Cre-reporter virus at PND 0 or PND 64. Tissue was prepared from animals 27 and 14 days after injection, respectively. Arrow points to a labeled PC in PND 0-injected cerebellum. Note the absence of Cre activity in PCs in mature animals. Scale bar in lower right applies to all images.

We chose to pursue *c-kit* as a candidate gene because a recent analysis of the transcriptomes of GABA-releasing cerebellar neurons indicated high selectivity for this gene in MLIs over PCs [17], and because c-Kit-GFP reporter lines showed evidence of MLI targeting [19, 20]. We used a knock-in, gene-targeting strategy to insert a *Cre* allele at the endogenous *c-kit* locus separated by an internal ribosome entry site (IRES) allowing for bicistronic transcript expression (Fig 1A). In a first approach to characterize Cre-expressing cell types, we bred *c-kit*^*IRES-Cre*^ mice to a Cre-reporter mouse line (Ai14; [21]). In tissue from *c-kit*^*IRES-Cre/+*^:Ai14 offspring (PND 36–38), we observed fluorescent cells throughout the cerebellum including abundant MLIs but also PCs (Fig 1B). This result was not surprising, however, because c-Kit expression has been observed in neonatal PCs and has been implicated in neural development [24] so its expression pattern is likely subject to change during neurogenesis and migration.

**Fig 2:**
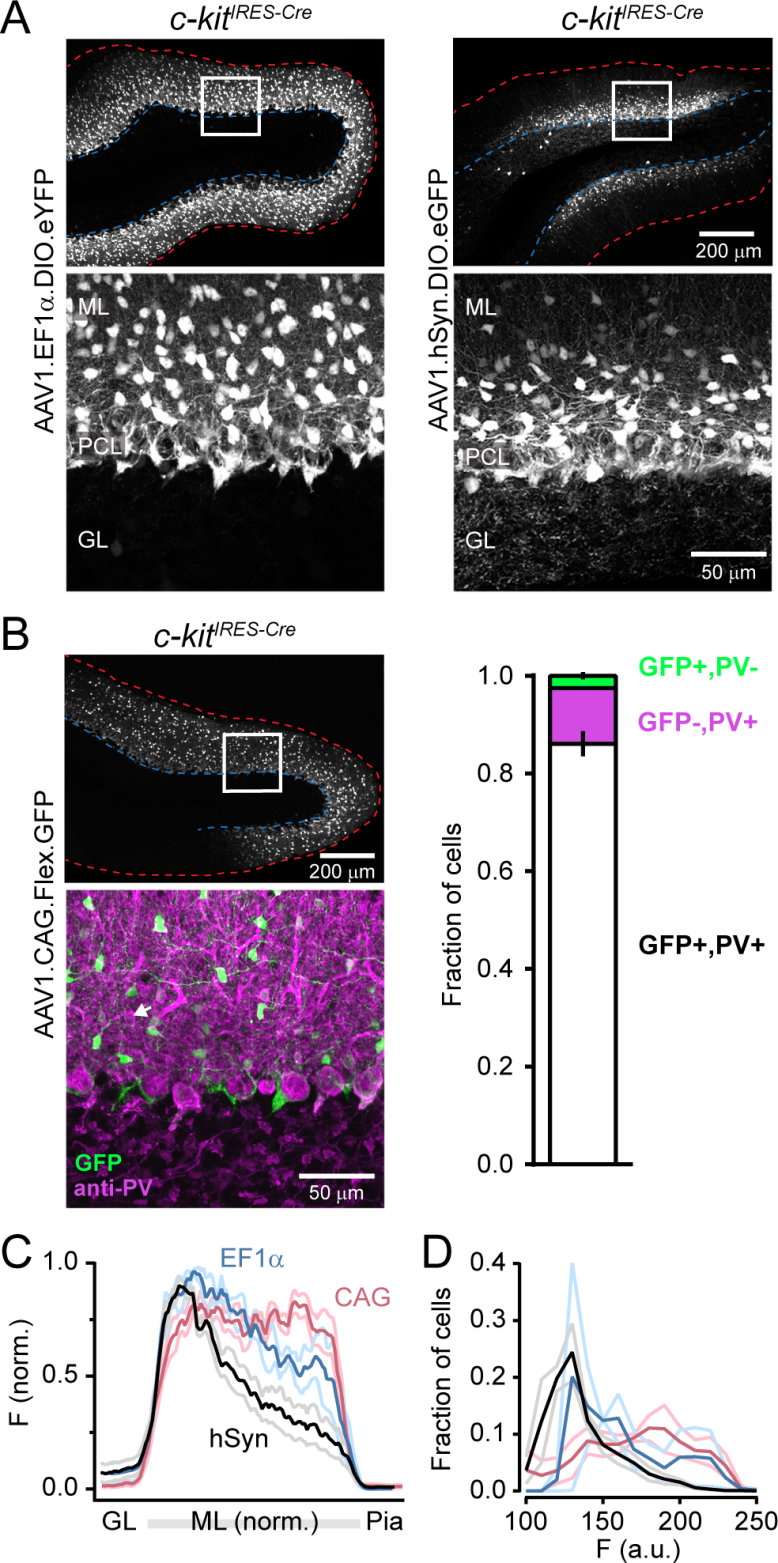
MLI transduction independent of viral promoter. **(A)** Tissue from *c-kit*^*IRES-Cre*^ mice injected with AAVs (≥PND 30) using either the EF1α or hSyn promoter. Scale bars apply to all images of equivalent size. Dashed red and blue lines indicate the pia and the PCL/GL boundary, respectively. **(B)** On the left, an image from *c-kit*^*IRES-Cre*^ mice injected with AAVs using CAG promoter and co-labeled with PV antibody (low magnification view of GFP label is shown above). Arrow marks PV+ cell without GFP. On the right, quantification of GFP and PV labeling across identified cells in the molecular layer (n = 8 sections from 2 mice). **(C)** The average intensity profile (with SEM) of fluorescence in the across cortical layers from *c-kit*^*IRES-Cre*^ mice injected with Cre-reporter AAVs using either hSyn (n = 6 sections from 2 mice), CAG (n = 5 sections from 2 mice), or EF1α (n = 3 sections from 2 mice) as promoters. Note that the ML fluorescence borders the GL due to the basket cell pinceaux. **(D)** Histogram of fluorescence intensity distributions for individual cells identified from images used for panel C.

To gain better insight into the developmental regulation of *c-kit* in the cerebellum, we injected AAV-containing Cre-dependent GFP into the vermi of *c-kit*^*IRES-Cre*^ pups shortly after birth (PND 0) and, after allowing time for expression, imaged tissue prepared from perfused animals. In these mice, we found sparse labeling of both MLIs and PCs (Fig 1C). In contrast, when we injected Cre-reporter AAV into the cerebella of mature mice (PND >30), we observed robust transduction of MLIs but Cre-dependent GFP expression in PCs was clearly absent (Fig 1C). This result persisted across three different viral promoters that are in common use (Fig 2A and 2B). Upon closer examination of the transduced cells within the molecular layer, we calculated a labeled cell density of ~1*10^5^/mm^3^ for both vermis and hemisphere injections (in agreement with published MLI densities [25]). We subsequently confirmed this population was nearly all interneurons using the marker protein PV (Fig 2B). On average 97 ± 1% of GFP-positive cells co-labeled with PV antibody. Notably, on inspection of tissue infected with AAV using the hSyn promoter, it was apparent that interneurons in the inner portion of the molecular layer were preferentially labeled indicating biased transduction of presumptive basket cells by viruses containing this *cis*-element as evidenced by the well-defined axon plexus of pinceaux surrounding PCs (Fig 2A). Importantly, we obtained more widespread, uniform MLI transduction in the molecular layer using AAV vectors incorporating CAG or EF1α as promoters, also without any PC labeling (Fig 2C and 2D).

**Fig 3:**
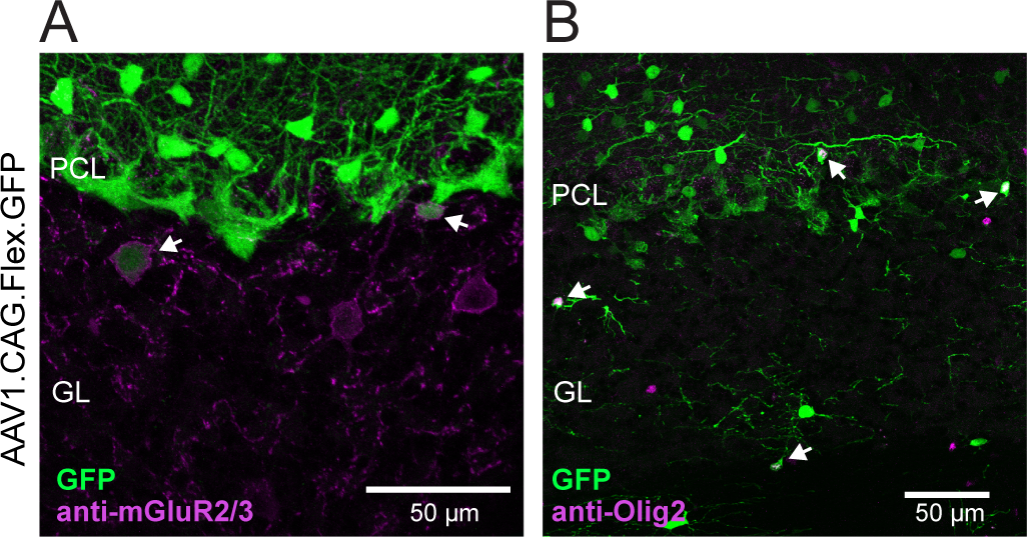
Sparse non-MLI targeting in *c-kit*^*IRES-Cre*^ mice. **(A)** Fluorescence image from a *c-kit*^*IRES-Cre*^ mouse injected with Cre-reporter virus showing sparse Cre-activity in GoCs as indicated by co-labeling for mGluR2/3. Arrowheads point to co-labeled cells. **(B)** Same as above but using an Olig2 antibody to label oligodendrocytes. Note that sparse, co-labeled cells are found in both the molecular layer (top two arrows) as well as the GrC layer (bottom two arrows).

It should be noted that, even in adults, we observed sparse labeling of some non-MLIs. To determine the identities of these labeled cells in the GrC layer we used staining for known cell marker proteins. It has been previously reported that c-Kit is expressed in GoC interneurons, though the expression level was not quantified [15]. Therefore, we used mGluR2/3 immunostaining to label putative GoCs [26] and observed partial overlap with a subset of virally transduced cells in this region (Fig 3A). On average 18.0 ± 7.1% of mGluR2/3-positive GoCs in the combined population of CAG-, EF1α-, and hSyn-injected mice were dually-labeled (mGluR2/3+ cells make up ~70% of total GoC population; [26, 27], and GoCs are the only interneuron cell type in the GrC layer immunoreactive for mGluR2/3 [27, 28]). While some GoC transduction was apparent for each of these three promoters, we did not specifically look for differences among them. This result did not vary across cerebellar regions (19% of cells in vermis and 16% of cells in hemisphere; n = 5 and 2 mice, respectively), though further investigation may be warranted regarding differences between individual lobules. Morphologically, a second population of transduced cells had the appearance of glia which we confirmed with immunohistochemistry using an antibody against Olig2, an oligodendrocyte marker protein (Fig 3B). Thus, although the *c-kit*^*IRES-Cre*^ line allows for the robust Cre-dependent viral transduction of MLIs independent of PCs (or GrCs) in mature mice, we did observe modest non-MLI targeting as expected provided *c-kit* mRNA expression by in situ hybridization found publically within the Allen Brain Atlas (http://mouse.brain-map.org/gene/show/16363).

### Functional evaluation of MLI targeting using optogenetics

**Fig 4:**
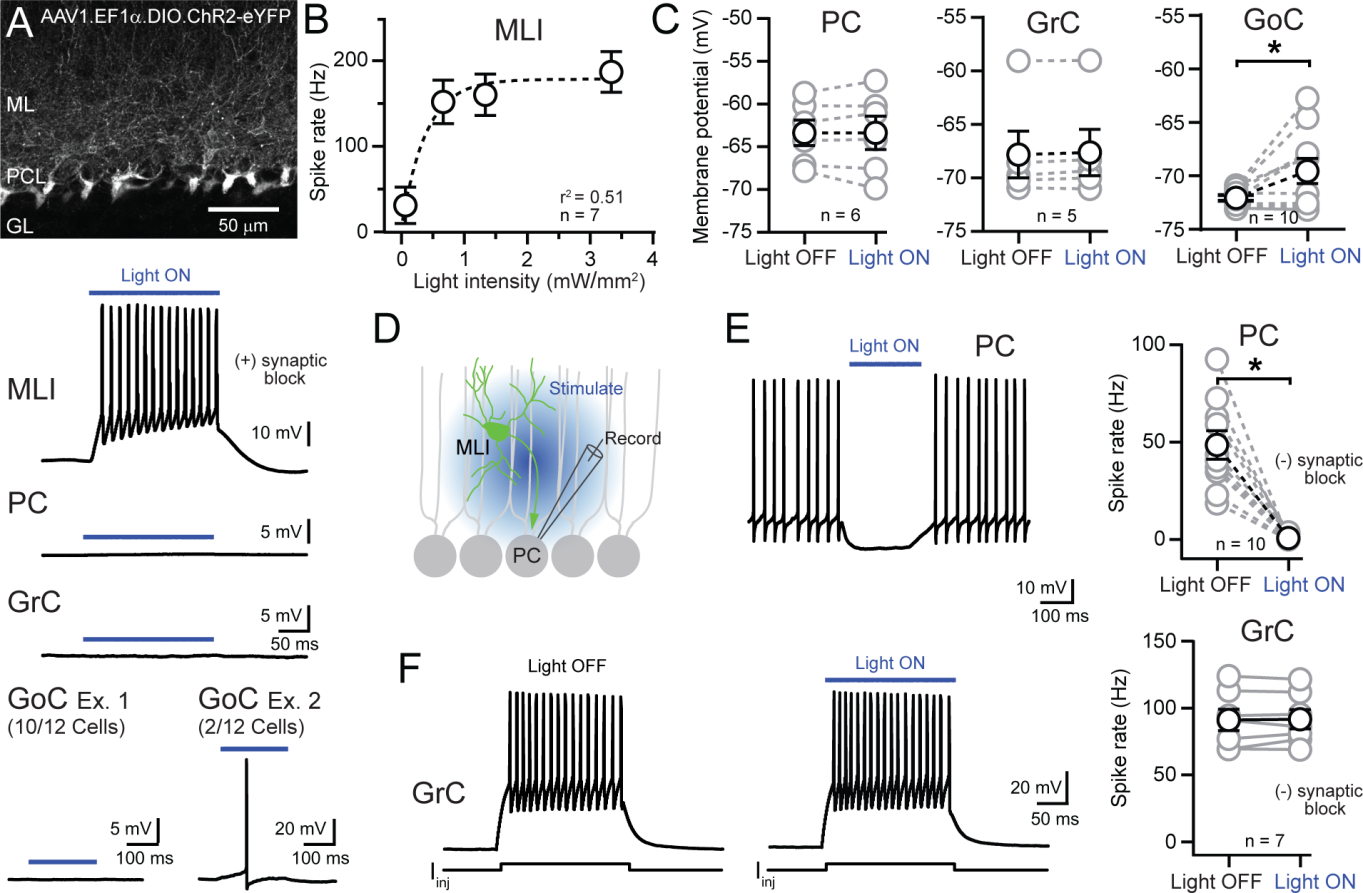
Functional evaluation of MLI transduction in c-kit^IRES-Cre^ mice using optogenetics. **(A)** Fluorescence image showing MLIs in a *c-kit*^*IRES-Cre*^ mouse transduced by AAV containing Cre-dependent ChR2. Below are representative traces from an MLI, PC, GrC, and two GoCs in a region of high ChR2 expression during illumination with 470 nm light. Recordings were performed in the presence of blockers for AMPA, NMDA, and GABA_A_ receptors. **(B)** Summary plot showing the relationship between spike frequency and light intensity in ChR2-expressing MLIs. A monoexponential function has been fit to the data. **(C)** Blue light illumination (3.3 mW/mm^2^) did not affect the membrane potential in either PCs or GrCs (P = 0.9861 and P = 0.1875) although there was a small effect in GoCs (*P = 0.03, paired t-tests). Synaptic inputs were blocked in all recordings. **(D)** Diagram depicting the PC recording configuration. MLIs expressing ChR2 were photo-stimulated using wide-field, blue-light illumination in the absence of GABA_A_ receptor block. **(E)** On the left, optogenetic stimulation during sustained firing in a PC. On the right, the effect of optogenetic MLI stimulation (3.3 mW/mm^2^) on the PC spike rate (*P = 0.0001, paired t-test). **(F)** Left, representative traces from a GrC, induced to spike by current injection, with and without blue light illumination. Summary plot on the right showing the absence of an optogenetic effect on GrC spiking (3.3 mW/mm^2^) (P = 0.7716, paired t-test).

In a separate set of experiments, we functionally evaluated the specificity of MLI transduction in *c-kit*^*IRES-Cre*^ mice using optogenetics. Mature animals (PND >30) were injected with AAV containing Cre-dependent channelrhodopsin (ChR2)-eYFP and, after allowing for onset of expression (3–4 weeks), acute cerebellar slices were prepared. From this tissue we made patch-clamp recordings from major cerebellar neuron types in areas displaying high expression levels of marker protein fluorescence. In the presence of synaptic blockers, optogenetic excitation (λ = 470 nm; 200 ms) evoked spiking in MLIs (15/15) achieving >100 Hz firing with < 4 mW/mm^2^ of light intensity (Fig 4A and 4B). However, intense optical stimulation (3.3 mW/mm^2^; 200 ms) failed to drive spiking in PCs (n = 11) or GrCs (n = 12) due to the complete absence of optically-induced depolarization in these cell types (Fig 4C). Rarely, optical stimulation drove spiking in randomly selected GoCs (n = 2 out of 12 cells tested; Fig 4A) consistent with expression in these cell as observed with our mGluR2/3 analysis. Small depolarizing potentials (~2 mV on average; Fig 4C) may result from electrical coupling of ChR2-induced excitation emanating from a limited population of Cre-expressing GoCs because they form a well-connected syncytium through gap junctions [29]. Together, these results functionally demonstrate the abundant expression of Cre-dependent transgenes in MLIs in *c-kit*^*IRES-Cre*^ mice.

MLIs provide powerful feed-forward inhibition onto PCs (Fig 4D) and are thus an important element involved in shaping cerebellar output [30]. We examined whether PC spiking could be inhibited by ChR2 activation of this local inhibitory circuit in *c-kit*^*IRES-Cre*^ mice. PCs that were held at a suprathreshold voltage displayed sustained firing at high rates as observed *in vivo* [31]. In the absence of synaptic blockers, whole-field excitation of MLIs (470 nm; 3.3 mW/mm^2^; 200 ms) completely abolished spiking in patched PCs (Fig 4E) demonstrating rapid and robust optogenetic control of PC excitability. That GoC axons synapse onto many hundreds of GrCs [32, 33] could indicate widespread divergence of their inhibition onto the cerebellar neural network. To determine whether the sparse activity of GoCs expressing ChR2 was richly represented in the GrC population, we measured for optically-induced voltage changes in randomly selected GrC with synaptic transmission unblocked. However, light failed to evoked IPSP-like responses (resting membrane potential 99.7 ± 0.2% of control; P = 0.1513; n = 4, paired t-test) or alter spiking elicited by current injection in GrCs (Fig 4F) which should be sensitive to both subtractive and divisive inhibition. Alternatively, dense electrical stimulation of presumptive GABA-releasing GoCs was sufficient to evoke hyperpolarizing IPSPs (-3.1 ± 1.7 mV; n = 3) and reduce GrC spiking [34] (73.7 ± 7.0 and 14.3 ± 9.5 Hz spiking; control and GoC stimulation, respectively; P = 0.007, n = 4, paired t-test) a result that was reversed by the GABA_A_ receptor antagonist SR95531 (20 μM; 84.0 ± 25.5 and 73.6 ± 10.9 Hz spiking; control and stimulation, respectively; P = 0.6103, n = 3; paired t-test). This observation is consistent with *in vivo* measurements showing that inhibition from GoC interneurons can significantly affect sustained GrC firing [35]. Together, these results indicate that sparse expression of ChR2 in GoCs is insufficient to modify widespread GrC activity in *c-kit*^*IRES-Cre*^ mice.

### Chemogenetic inhibition of MLIs

**Fig 5:**
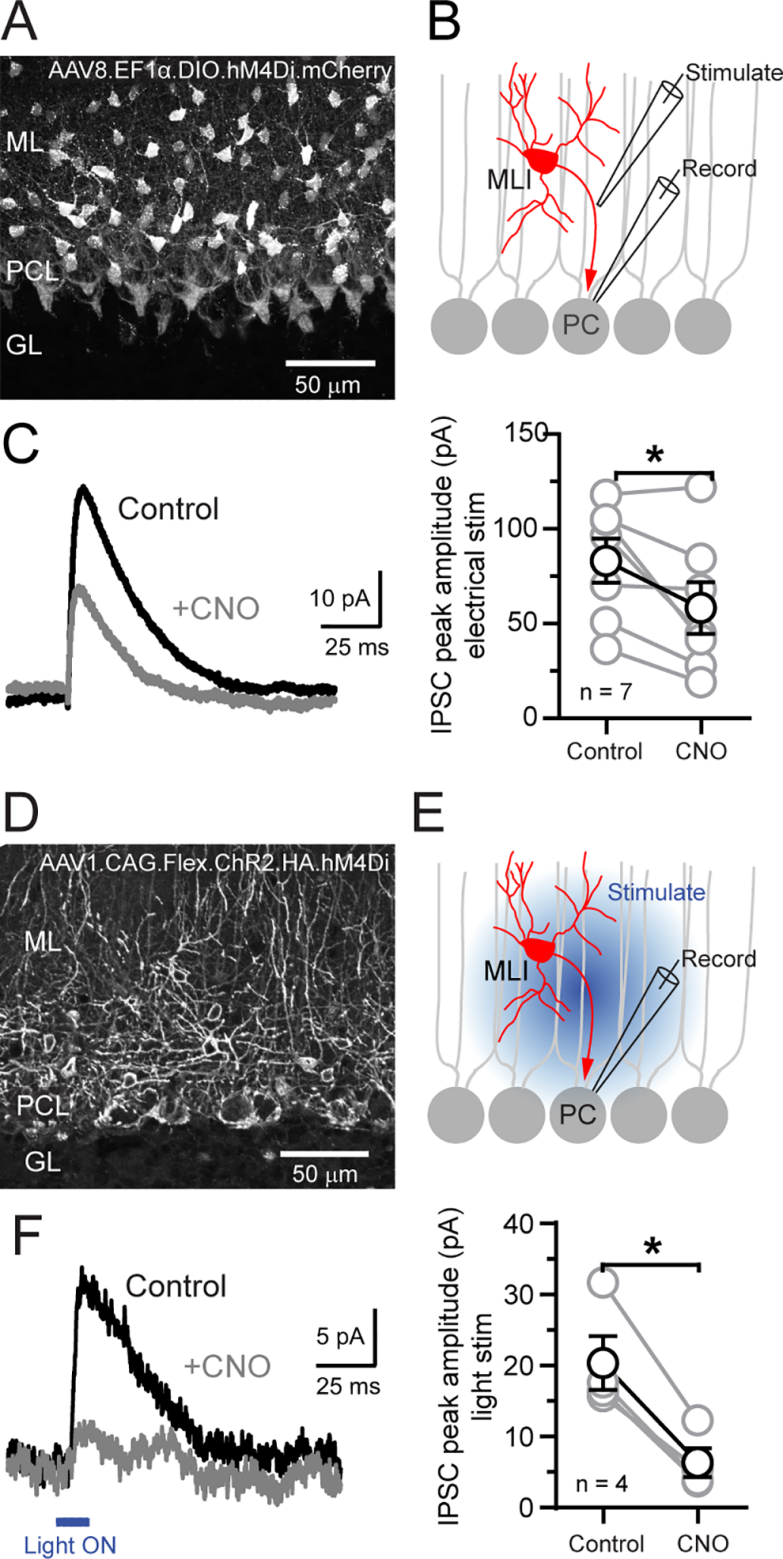
Chemogenetic suppression of molecular layer inhibition using DREADDs. **(A)** MLIs in a *c-kit*^*IRES-Cre*^ mouse transduced with the DREADD hM4Di by Cre-dependent AAV. **(B)** Cartoon depicting the PC recording configuration. MLIs in a region of high hM4Di expression were induced to fire by extracellular electrical stimulation in the molecular layer. **(C)** Average IPSCs recorded in a PC in control and after application of CNO. Summary plot on the right shows the effect of DREADD activation on the peak IPSC amplitude in PCs (*P = 0.035, paired t-test).**(D)** MLIs in a *c-kit*^*IRES-Cre*^ mouse transduced with a bicistronic, Cre-dependent AAV containing both ChR2 and hM4Di. **(E)** Cartoon depicting the PC recording configuration. MLIs were stimulated using blue light directed towards the molecular layer. **(F)** Similar to panel C but using optogenetic (3.3 mW/mm^2^) rather than electrical stimulation to drive MLI inhibition onto PCs (*P = 0.005, paired t-test).

The toolkit for genetically-encoded effectors of neural activity is rapidly expanding, building on light-based opsin technologies [36] as well as engineered designer receptors [37]. To examine the utility of our *c-kit*^*IRES-Cre*^ driver line, we conditionally expressed the inhibitory DREADD receptor hM4Di [38] in MLIs by viral infection (Fig 5A). In acute cerebellar slices prepared from injected mice, we recorded IPSCs in PCs elicited by direct electrical stimulation of MLIs (Fig 5B). Bath application of the selective cognate ligand, clozapine-N-oxide (CNO; 0.2–1 μM; ≥15 min), reduced the amplitude of evoked IPSCs (Fig 5C). It may be that hM4Di had a partial effect on silencing MLI activity due to the intensity of electrical stimulation overcoming the modest hyperpolarization induced by hM4Di [39]. Therefore, in a similar experiment, we instead transduced MLIs with a bicistronic construct containing both hM4Di and ChR2 and used optogenetic stimulation to drive inhibitory MLI transmission onto PCs (Fig 5E). In this case, hM4Di-induced silencing was nearly complete (Fig 5F) indicating that DREADDs can potently suppress MLI neurotransmission. Together these findings demonstrate that the *c-kit*^*IRES-Cre*^ mouse allows for functional interrogation of the MLI circuit through both optogenetic- and chemogenetic-based approaches.

## Discussion

In this report, we used the endogenous transcriptional machinery of *c-kit* to genetically distinguish MLIs from other major cell types in the cerebellum. The use of *c-kit*^*IRES-Cre*^ mice enabled the expression of marker proteins as well as genetically encoded effectors in MLIs, independent of PCs and GrCs, allowing for their activation or inactivation, thus demonstrating the flexibility of this mouse line and potential utility for *ex vivo* and *in vivo* circuit analysis experiments.

Our motivation was to gain genetic access to MLIs free of many of the restrictions associated with previously published methods. For example, although transgene expression in MLIs was achieved in wildtype mice by AAV transduction under control of the hSyn promoter, it also resulted in expression in candelabrum cells, Golgi cells, Lugaro cells, and to a lesser extent PCs [25]. Improvements were made by use of Cre-dependent AAV-hSyn in *PV-Cre* mice with purported targeting of MLIs [40]. However, given that PV is well-expressed by PCs it remains highly probable that low-level expression also occurred in this cell type as indicated by faint reporter gene fluorescence in this report. Low-level PC transduction may be acceptable for many applications, however, it is unlikely that use of other promoters (e.g., CAG or EF1α) in AAV could achieve similar selectivity in this combinatorial approach. We believe this is a restriction and limits experimental flexibility, especially given the possibility that hSyn is biased towards basket cells, based on our observations.

Using AAV vectors without regard to promoter type, we could introduce Cre-dependent transgenes in most MLIs within an area near the site of injection, in either vermis or hemisphere regions, and generate robust and efficient protein expression in this cell type. As assessed anatomically and functionally, Cre activity was absent in both PCs and GrCs and was minimally apparent in a minority of GoCs and a sparse population of oligodendrocytes as expected for the endogenous expression pattern of c-Kit in mice. Importantly, we observed evidence for the embryonic promotion of *c-kit* in PCs. Thus our *c-kit*^*IRES-Cre*^ line will not provide selective experimental access to MLIs if recombination occurs during embroyogenesis although it is possible that intersectional strategies based on two or more genes will offer the potential for improved specificity during the course of development [41]. Lastly, and perhaps most importantly, Cre expression was not perfectly exclusive to MLIs in *c-kit*^*IRES-Cre*^ mice and therefore the consequence of some Golgi cell targeting must be accounted for when using these mice.

Previously, a transgenic mouse with Cre under the regulation of *c-kit* has been developed [42]. However, Cre-reporter labeling was observed in the hippocampus and retina but not in the cerebellum. This is surprising considering the overwhelming evidence for c-Kit expression in the cerebellar molecular layer [15–18] and the presence of GFP in MLIs of c-Kit-GFP mice [19, 20]. That the previous mouse was engineered using a partial fragment of the *c-kit* promoter could result in inadvertent targeting and therefore not reliably recapitulate the endogenous *c-kit* gene expression profile. Recently, a knock-in mouse line using a *CreER*^*T2*^ allele under control of *c-kit* (*c-kit*^*CreERT2*^) has been engineered allowing for inducible control of Cre activation following administration of tamoxifen [43, 44]. By providing temporal regulation, this inducible Cre line may present a significant advantage over *c-kit*^*IRES-Cre*^ mice if genetic manipulation is desired specifically in MLIs without the use of viruses by restricting the activity of Cre during the developmental period when Cre is active in embryonic PCs. However, the expression profile of Cre has not been reported in the brains of *c-kit*^*CreERT2*^ mice nor has the efficiency of recombination been assessed in various cerebellar cell classes following tamoxifen induction. Further investigation will therefore be required to know whether this *c-kit*^*CreERT2*^ mouse line will be a useful tool for genetic manipulations in MLIs. One important caveat with *c-kit* Cre knock-in lines is the potential for haploinsufficiency problems. For example, loss of normal c-Kit function leads to multiple abnormalities and sterility [45, 46]. Importantly, our mouse has no obvious health problems or phenotype. Homozygous mice successfully breed, lack white-spotting syndrome (as seen with deletions of *c-kit*; *[47]*), and the cerebellum appears structurally normal. However, we did not directly test for any changes in c-Kit expression levels in our line.

We found that the *c-kit*^*IRES-Cre*^ line allowed for the widespread transduction of MLIs throughout the molecular layer when paired with Cre-dependent AAVs using high-efficiency promoters. However, when using the hSyn promoter, we noted the appearance of biased labeling of MLIs in the inner portion of the molecular layer where a majority of pinceaux projecting basket cells reside [48, 49]. This finding was consistent across the one to four week expression time we studied. We caution that it is unlikely that we specifically differentiated interneuron types in the molecular layer using hSyn as there was clear transduction in some superficial cells (presumably stellate cells). Although we speculate that hSyn may be more active in basket cells relative to stellate cells, other strategies may be better suited for MLIs subtyping [17]. For example, *Ascl1*^*CreER*^ mice may be useful for this purpose [50]. However, because Ascl1 helps control the balance of glia types, dense targeting of MLIs without Cre activity in glia may be difficult to achieve as both of these cell types share a common lineage [50, 51]. Notably, we also observed Cre activity in some glial cells in *c-kit*^*IRES-Cre*^ mice which was more pronounced during development but quite limited in adulthood.

When deciding on how best to target MLIs, we also considered other genes with potentially promising expression profiles, but selected *c-kit* for a variety of reasons. For example, *Nos1* that encodes nNOS has been used to selectively target ChR2 expression to MLIs in BAC transgenic mice [52, 53]. Furthermore, *Nos1-Cre* knock-in mice have been used to selectively express optogenetic tools in MLIs [54]. However, nNOS is found in GrCs—although at lower levels than in MLIs [8–10]. Given that GrCs are the most numerous neurons in the brain, expressing even a small amount of the Cre enzyme using a knock-in approach to recapitulate endogenous expression patterns could be problematic, particularly for alternate forms of Cre-based interrogation of neural circuit function other than optogenetics. Other alternative approaches have relied on the ability of the hSyn promotor to preferentially drive transgene expression in interneurons over PCs [25, 40]. Nonetheless, any resulting PC expression, even at lower levels [25], could lead to PC activity perturbations with some effectors. In practice, a complimentary approach using multiple lines such as *c-kit*, *Nos1*, or others (once validated; [55–57]) to study MLIs could overcome the individual limitations of each line; viral strategies may also be beneficial in this respect [58].

In summary, the *c-kit*^*IRES-Cre*^ mouse line should prove to be a valuable tool for studying the function of MLIs in adult mouse cerebellum as demonstrated recently [58] (see this report for additional images of Cre-dependent transgene expression in inhibitory interneurons). The utility of the Cre/lox system allows for enormous experimental flexibility accommodating the rapid advancement of genetically encoded calcium indicators, voltage indicators, optogenetics, chemogenetics, and other genetic tools through viral delivery with little constraint. Therefore *c-kit*^*IRES-Cre*^ mice maybe advantageous over stably expressing lines for individual reporters or actuators of activity that are time-consuming to build and costly to produce. It also remains possible that conditional gene inactivation could be accomplished in MLIs by Cre-dependent viral delivery of interference RNA technologies or guide RNA if used in conjunction with the CRISPR/Cas9 system. However, this is yet to be experimentally verified.

## Supporting information

S1 File Supporting data. (XLSX)
